# Identification of a FOXP3^+^CD3^+^CD56^+^ population with immunosuppressive function in cancer tissues of human hepatocellular carcinoma

**DOI:** 10.1038/srep14757

**Published:** 2015-10-06

**Authors:** Xiaofeng Li, Jirun Peng, Yanli Pang, Sen Yu, Xin Yu, Pengcheng Chen, Wenzhen Wang, Wenling Han, Jun Zhang, Yanhui Yin, Yu Zhang

**Affiliations:** 1 Department of Immunology, Peking University Health Science Center, Beijing 100191, China; 2 Department of Molecular Imaging and Nuclear Medicine, Tianjin Medical University Cancer Institute and Hospital, National Clinical Research Center for Cancer, Key Laboratory of Cancer Prevention and Therapy, Tianjin 300060, China; 3 Center of Hepatobiliary Surgery, Peking University People’s Hospital, Beijing 100044, China

## Abstract

The liver resident lymphoid population is featured by the presence of a large number of CD3^+^CD56^+^ cells referred as natural T cells. In human hepatocellular carcinoma (HCC) patients, the natural T cells were found to be sharply decreased in tumor (5.871 ± 3.553%) versus non-tumor (14.02 ± 6.151%) tissues. More intriguingly, a substantial fraction of the natural T cells (22.76 ± 18.61%) assumed FOXP3 expression. These FOXP3-expressing CD3^+^CD56^+^ cells lost the expression of IFN-γ and perforin, which are critical for the effector function of natural T cells. On the other hand, they acquired surface expression of CD25 and CTLA-4 typically found in regulatory T (Treg) cells. Consistent with the phenotypic conversion, they imposed an inhibitory effect on anti-CD3-induced proliferation of naive T cells. Further studies demonstrated that transforming growth factor β1 (TGF-β1) could effectively induce FOXP3 expression in CD3^+^CD56^+^ cells and the cells were thus endowed with a potent immunosuppressive capacity. Finally, Kaplan-Meier analysis revealed that the relative abundance of FOXP3-expressing CD3^+^CD56^+^ cells in tumor tissues was significantly correlated with the survival of HCC patients. In conclusion, the present study identified a new type of regulatory immune cells whose emergence in liver cancer tissues may contribute to tumor progression.

Hepatocellular carcinoma (HCC) is one of the most common malignancies worldwide, ranking fifth in prevalence and third in mortality^1^. Infections with the hepatitis B or C virus constitute a major risk factor for HCC. The viral infection induces chronic inflammation and liver cirrhosis and HCC lesions eventually arise in the inflamed and cirrhotic environment. Due to the difficulty in early diagnosis and the limited therapeutic options, the 5 year overall survival remains low at 18%^2^. Thus, there is an urgent need for new therapeutic options for HCC at advanced stages. An emerging and very promising approach is immunotherapy^3^,^4^, which has gained increasing momentum in recent years as immune checkpoint blockade with anti-CTLA4 or anti-PD-1 antibodies and chimeric antigen receptor T-cell therapy demonstrate clear evidence of objective responses^5^. In a recent study, systemic therapy of patients with chronic hepatitis C infection and HCC with the CTLA-4 inhibitory antibody tremelimumab was both safe and efficacious resulting in partial responses and high disease control rates^6^. To further improve the efficacy of cancer immunotherapy, a detailed understanding of the role of the immune system in the development and control of HCC lesions is required.

Mounting evidence highlights a complex role of the immune system in the development and progression of HCC. An intratumoral accumulation of lymphocytes was detected in some patients and the infiltration of T cells and especially cytotoxic CD8^+^ T cells was found to be a good prognostic factor^7^,^8^. Moreover, a number of studies documented spontaneous humoral and cellular immune responses to a variety of tumor-associated antigens in HCC patients^3^,^9^,^10^. A strong CD8^+^ T cell response against several tumor-associated antigens was shown to coincide with improved survival^11^. Furthermore, it has been repeatedly reported that concomitant activation of the immune system contributes to the therapeutic effects of conventional treatments such as surgical resection, locoregional therapy and chemotherapy^3^. In most cases, however, the anti-tumor immunity is apparently not sufficient to control the tumors. This failure is primarily due to the multiple passive and active mechanisms employed by tumor to evade the host’s immune attack. The active inhibition of immune responses is mainly mediated by the various immune suppressor cells present in tumor tissues, such as regulatory CD4^+^ T cells (Tregs), myeloid derived suppressor cells (MDSCs), and tumor-associated macrophages (TAM)^12^.

Treg is believed to play a critical role in tumor immune evasion^13^. Its increase has been reported in a wide array of human malignancies, including HCC^14^,^15^. The study by Fu *et al*. demonstrated that an up-regulation of Tregs was associated with a significantly reduced CD8^+^ T cell infiltration of tumors and predicted a worse outcome for HCC patients^16^. MDSC, a heterogenous population of myeloid origin, constitutes another mechanism of immunosuppression^17^. The frequency of CD14^+^HLA-DR^low/neg^ MDSC is strongly increased in HCC patients^18^, for which the activated hepatic stellate cells are speculated to be a major inducer^19^. In addition to an inhibitory effect on autologous T cell proliferation, CD14^+^HLA-DR^low/neg^ cells are capable of converting CD4^+^ T cells into Tregs in co-cultures^18^. TAM is also an important player in tumor-induced immune suppression and a high frequency of infiltrating TAMs is often associated with poor prognosis^20^. Zheng and his colleagues demonstrated that the activated macrophages in the peritumoral stroma exerted suppressive functions either through PD-L1 expressed on their surface or via expansion of memory Th17 cells^21^,^22^. More recently, Han *et al*. reported the identification of a novel subset of CD14^+^CTLA-4^+^ regulatory dendritic cells in HCC patients, which suppresses antitumor immune response through CTLA-4-dependent IL-10 and indoleamine-2,3-dioxygenase (IDO) production^23^. It will not be surprising if there exist additional subsets of tumor-related regulatory cells in HCC, especially in view of the unique distribution of the hepatic lymphoid population.

The composition of lymphocyte in the liver differs markedly from that in the peripheral blood. In addition to a high proportion of natural killer (NK) cells (up to 50% of the total hepatic lymphoid cells), the liver is populated by significant numbers of “unconventional” T cells which are absent or present at very low levels in the blood, such as T cells that express both CD3 and CD56, T cells that are either double positive or double negative for CD4 and CD8, and T cells that express the CD8αα homodimer rather than the CD8αβ heterodimer^24^,^25^. Together, they form a unique microenvironment, which may impose a profound impact on immune responses in the liver. Of particular interest are CD3^+^CD56^+^ cells, which account for less than 2% of peripheral T cells but about one third of hepatic T cells^26^. These cells not only exhibits dual T cell and natural killer cell phenotype, but also demonstrate similar functionality to natural killer T (NKT) cells. As such, they are capable of both cytokine-induced killing of K562 cells and TCR-mediated cytotoxicity against P815 cells and rapidly produce high levels of Th1 and Th2 cytokines upon pharmacological stimulation or CD3 cross-linking^27^. The majority of these cells, however, lack the expression of Vα24 TCR characteristic of invariant NKT cells. To reflect this fact, the hepatic CD3^+^CD56^+^ cells are also called “natural” T (NT) cells^26^.

The function of NT cells remains ill-defined. Given the high prevalence of these cells in the liver, it is reasonable to speculate that they play an active role in the immune homeostasis of normal liver and their perturbation may contribute to the pathogenesis of liver diseases. We were specifically interested in their implication in the pathology of HCC. The present study detected a large number of FOXP3-expressing cells in an overall much reduced NT compartment in liver cancer tissues. This newly identified subset of cells was subsequently characterized in terms of phenotypic and functional features and further investigated for a potential correlation with clinical outcomes.

## Results

### Reduced CD3^+^CD56^+^ natural T cells in TILs of HCC patients

**Figure 1:**
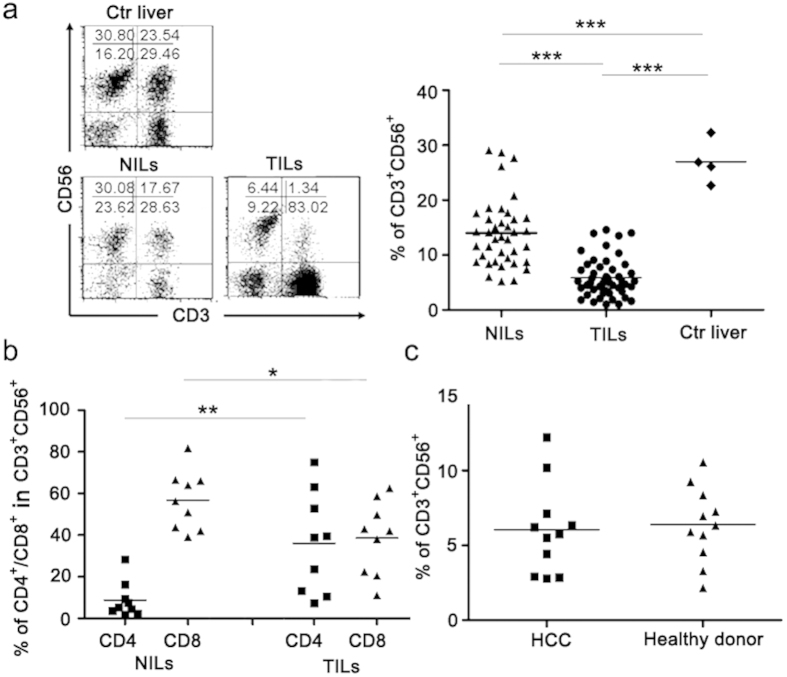
The markedly reduced CD3^+^CD56^+^ cell population and the over-representation of CD4^+^ cells in this population in tumor tissues of HCC patients. Tissue infiltrating lymphocytes were isolated from tumorous (TILs, n = 48) and adjacent non-tumorous (NILs, n = 39) tissues of HCC as well as liver tissues derived from hepatic benign hemangioma (Ctr liver, n = 4), and stained for CD3 and CD56 expression. (**a**) A representative FACS profile is shown on the left and the percentages of CD3^+^CD56^+^ cells in individual patients are summarized in the right. (**b**) Increased representation of CD4^+^ cells in the CD3^+^CD56^+^ cell population in TILs versus NILs. (**c**) Unaltered representation of CD3^+^CD56^+^ cells among PBMCs of HCC patients in comparison with healthy donors (n = 11). ^*^P < 0.05, ^**^P < 0.01, ^***^P < 0.001.

Previous studies from our group and others have revealed remarkable differences in the composition of the lymphoid cells infiltrating tumor versus non-tumor tissues^28^,^29^. The present study was focused on the CD3^+^CD56^+^ population, which is sometime referred as “natural T cell” to distinguish it from the classical natural killer cell^26^. This population accounted for 1/4-1/3 of liver resident lymphoid cells in hepatic benign hemangioma patients which are believed to have virtually normal liver tissues. In contrast, its percentage dropped to a single digit (5.871 ± 3.553%, n = 48) in TILs from HCC patients (Fig. 1a). Of note, a significant, although less dramatic reduction of CD3^+^CD56^+^ cells was also observed in adjacent non-cancerous tissues (14.02 ± 6.151%, n = 39) (Fig. 1a), which is probably related to the inflammatory conditions commonly seen in the liver of HCC patients. While the majority of CD3^+^CD56^+^ cells in NILs were CD8^+^, they were equally split into CD4^+^ and CD8^+^ subsets in TILs (Fig. 1b). We did not analyze the distribution of CD4/CD8 in CD3^+^CD56^+^ T cells in normal liver in our study. However, there were several reports on this issue^26^,^30^, and, the distribution of CD4/CD8 in natural T cells (CD3^+^CD56^+^) in adjacent non-cancerous tissues of HCC patients in our study (n = 9, 8.75 ± 2085 *vs* 56.70 ± 4.71) was very close to the reported results. Despite the reduced representation in the liver, the percentage of CD3^+^CD56^+^ cells in PBMCs was comparable between HCC patients and healthy donors (Fig. 1c), indicating that it is likely the consequence of altered local microenvironment rather than a systemic effect.

### Identification of FOXP3^+^CD3^+^CD56^+^ cells in TILs from HCC patients

**Figure 2:**
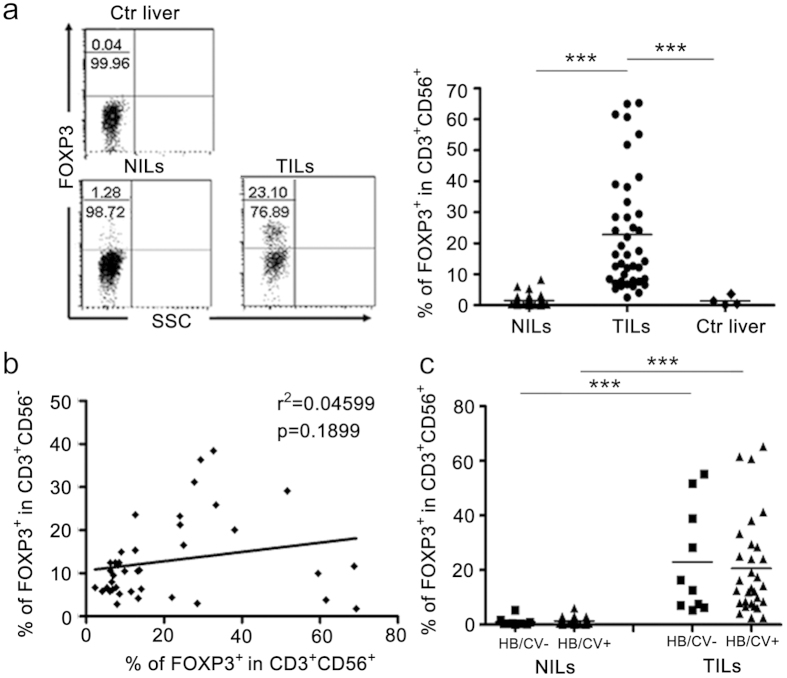
The presence of FOXP3^+^CD3^+^CD56^+^ cells in HCC tumor tissues. FOXP3 expression was determined by flow cytometry in CD3^+^CD56^+^ cells from control liver tissues (n = 4), tumorous (n = 41) and non-tumorous (n = 37) tissues of HCC patients. (**a**) A representative FACS profile is shown on the left and the percentage of FOXP3^+^ cells in CD3^+^CD56^+^ cells from individual patients are summarized in the right. The numbers denote the percentage of the cell population in the left two quadrants. (**b**) A correlation analysis was performed between the percentage of FOXP3^+^ cells in the CD3^+^CD56^+^ cell population and that in CD3^+^CD56^–^ conventional T cell population in the TILs from 41 HCC patients. (**c**) FOXP3 expression in CD3^+^CD56^+^ cells in TILs and NILs were compared between HCC patients with or without a history of HBV/HCV infection.

In addition to the reduced cell number, a substantial fraction (22.76 ± 18.61%) of the CD3^+^CD56^+^ cells in liver cancer tissues acquired FOXP3 expression (Fig. 2a), a transcription factor critical for the development and function of conventional regulatory T cells^13^. In comparison, FOXP3^+^ cells were rarely detected in the CD3^+^CD56^+^ population from adjacent non-cancerous tissues and were completely absent from normal liver tissues (Fig. 2a). Here, it is necessary to point out that, intracellular staining for FOXP3 expression needed more cells than cell surface staining, thus only the tumor samples with sufficient isolated tumor infiltration lymphocytes were analyzed for the FOXP3 expression, and samples with limited isolated lymphocytes were only analyzed for the surface expression of CD3 and CD56. Given the accumulation of conventional regulatory T cells in HCC patients^14^,^15^,^16^, one may wonder whether the increased presence of FOXP3^+^CD3^+^CD56^+^ cells simply resulted from the acquisition of CD56 expression by FOXP3^+^CD3^+^CD56^–^ Treg cells. This speculation, however, appeared to be at odds with the apparent lack of correlation between the percentage of FOXP3^+^ cells in the CD3^+^CD56^+^ cell population and that in CD3^+^CD56^–^ conventional T cell population in the TILs (Fig. 2b). As hepatitis B/C virus infection are the principal causes of HCC in China^1^, we further compared FOXP3 expression in CD3^+^CD56^+^ cells in HCC patients with or without a history of viral infection. No significant difference was observed between the two groups (Fig. 2c), indicating that FOXP3 expression in CD3^+^CD56^+^ cells was not the consequence of previous viral infection.

### Phenotypic and functional characterization of the FOXP3^+^CD3^+^CD56^+^ cells in liver cancer tissues

**Figure 3:**
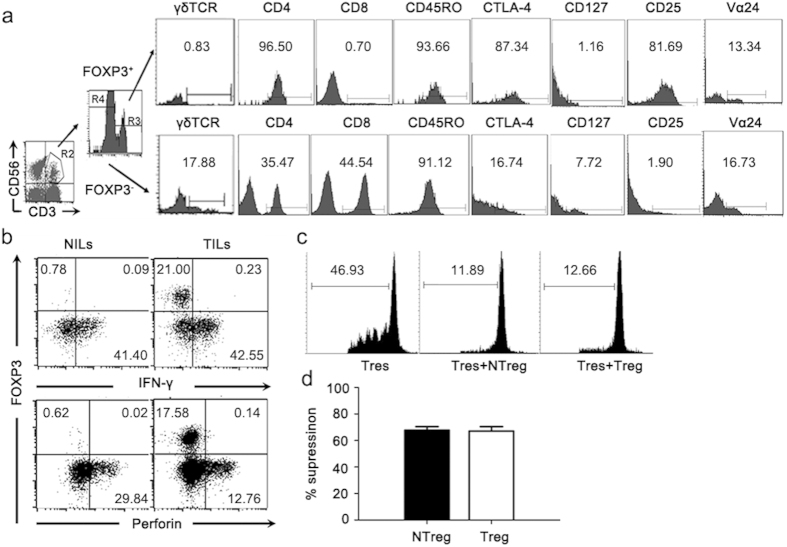
Comparative analyses of FOXP3^+^CD3^+^CD56^+^ cells versus CD3^+^CD56^+^FOXP3^–^ cells and the immunosuppressive function of FOXP3-expressing CD3^+^CD56^+^ cells in tumor tissues of HCC patients *in vitro*. Analyses were repeated with samples from 8–10 HCC patients with similar results. Data from a representative patient is shown. (**a**) CD3^+^CD56^+^ cells in TILs were divided into FOXP3^+^ and FOXP3^–^ subsets and analyzed for surface expression of γδ TCR, CD4, CD8, CD45RO, CTLA-4, CD127, CD25 and Vα24. (**b**) TILs or NILs from HCC patients were stimulated with Phorbol-12-myristate-13-acetate (50 ng/ml) and ionomycin (1 μg/ml) for 4 hours. FOXP3 and IFN-γ or Perforin expression in CD3^+^CD56^+^ cells were subsequently analyzed by flow cytometry. The numbers in the dot plots indicate the percentages of the corresponding subsets. (**c**) The anti-proliferative effect of tumor infiltrating FOXP3^+^CD3^+^CD56^+^ cells on CD4^+^CD25^–^ responder T cells. FOXP3^+^CD3^+^CD56^+^ cells were isolated and purified from the TILs of HCC patients and the CD4^+^CD25^-^ responder T cells were from autologous PBMC. Histograms represent CFSE dilution of labeled autologous CD4^+^CD25^–^ responder T cells cultured alone (Ctr) or at a 2: 1 ratio with Treg cells (Treg) or FOXP3^+^CD3^+^CD56^+^ cells (NTreg). Percentages of responder T cell proliferation are indicated. Data are representative of three independent experiments from three individual HCC patients. (**d**) The comparison of the proliferation-inhibitory effect of FOXP3^+^CD3^+^CD56^+^ cells (NTreg) with Treg cells from three HCC donors. The inhibitions of proliferation were normalized to proliferation of responder cells alone.

To our knowledge, it was the first report of FOXP3-expressing CD3^+^CD56^+^ T cells arising under pathological conditions. Their phenotypic and functional characters were subsequently investigated. The FOXP3^+^ cells were exclusively identified in the CD4^+^ subset of the CD3^+^CD56^+^ population and were deprived of γδ TCR^+^ cells. Similar to the conventional Treg cells, FOXP3^+^CD3^+^CD56^+^ cells expressed high levels of CD25 and CTLA-4 but no CD127, the α chain of IL-7 receptor (Fig. 3a).

Like NK cells, the CD3^+^CD56^+^ cells in the liver are good producers of IFN-γ and perforin^26^,^27^. TILs and NILs were stimulated with Phorbol-12-myristate-13-acetate (50 ng/ml) and ionomycin (1 μg/ml) and examined for the expression of IFN-γ and perforin in CD3^+^CD56^+^ cells in the context of FOXP3 expression. Notably, the expression of FOXP3 and IFN-γ/perforin was mutually exclusive (Fig. 3b). The acquisition of FOXP3, therefore, appeared to be accompanied with the loss of classical functions of natural T cells.

Next, we examined whether the cells were endowed with a suppressive activity upon FOXP3 expression. As indicated above, the majority of FOXP3^+^CD3^+^CD56^+^ cells were CD25^+^, whereas CD25 expression was hardly detected in FOXP3^–^CD3^+^CD56^+^ cells. Therefore, we used CD25 as a surrogate marker to obtain FOXP3-expressing CD3^+^CD56^+^ cells from TILs of HCC patients. To test their immunosuppressive function, purified FOXP3^+^CD3^+^CD56^+^ cells were added to the cultures of CFSE labeled autologous CD4^+^CD25^-^ T cells stimulated with anti-CD3. Results from CFSE dilution assay revealed a significant inhibitory effect of FOXP3^+^CD3^+^CD56^+^ cells on anti-CD3-induced proliferation of CD4^+^CD25^–^ T cells, and such an effect was comparable to that mediated by conventional Treg cells (Fig. 3c,d).

### Induction of FOXP3^+^CD3^+^CD56^+^ cells *in vitro*

**Figure 4:**
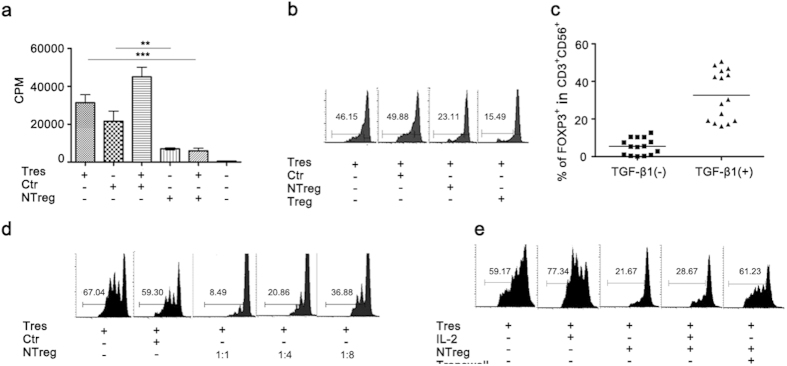
The induction and immunosuppressive function of FOXP3-expressing CD3^+^CD56^+^ cells *in vitro*. (**a**) *In vitro* expanded CD3^+^CD56^+^ cells were transduced with FOXP3-expressing lentiviral particles pCCL.FP3 or empty particles pCCL. The transduced cells were added at a ratio of 1:2 to cultures of autologous CD4^+^ T cells (Tres), and tested for inhibitory effect on cell proliferation induced by soluble anti-CD3 (1 μg/ml) in the presence of APCs using ^3^H thymidine incorporation. Data from three independent experiments are presented as Mean ± SD. Ctr: CD3^+^CD56^+^ cells transduced with control pCCL lentiviral particles; NTreg: CD3^+^CD56^+^ cells transduced with pCCL.FP3 lentiviral particles. (**b**) The anti-proliferative effect of FOXP3 transduced CD3^+^CD56^+^ cells was compared with that of CD4^+^CD25^high^ conventional Treg cells by monitoring CFSE dilution of responder cells. Representative results from one out of three independent experiments are presented. The numbers denote the percentages of cells which divided at least once. Ctr: CD3^+^CD56^+^ cells transduced with pCCL lentiviral particles; NTreg: CD3^+^CD56^+^ cells transduced with pCCL.FP3 lentiviral particles. (**c**) Induction of Foxp3 expression by TGF-β1. CD3^+^CD56^+^ cells isolated from NILs of HCC patients were stimulated with soluble anti-CD3 (1 μg/ml), anti-CD28 (1 μg/ml) and IL-2 (10 ng/ml) in the presence or absence of TGF-β1 (10 ng/ml). FOXP3 expression was detected by intracellular staining at day 4. The horizontal bar denotes the average of FOXP3^+^ cells in the cultures. (**d**) The anti-proliferative effect of TGF-β1-induced FOXP3^+^CD3^+^CD56^+^ cells (NTreg) were tested using CFSE dilution assay at a ratio of 1:1, 1:4, or 1:8 with autologous CD4^+^ T cells treated with anti-CD3. Ctr: uninduced CD3^+^CD56^+^ cells; NTreg: TGF-β1-induced CD3^+^CD56^+^ cells. (**e**) The inhibitory effect of TGF-β1-induced CD3^+^CD56^+^ cells (NTreg) on anti-CD3 induced proliferation of CD4^+^ T cells in the presence of exogenous IL-2 or in transwell cultures. The experiments were repeated three times with similar results.

To verify the immunosuppressive activities of FOXP3-expressing CD3^+^CD56^+^ cells, CD3^+^CD56^+^ cells expressing exogenous FOXP3 were generated through lentiviral transduction. CD3^+^CD56^+^ cells from NILs were first expanded *in vitro* to obtain sufficient numbers of cells. Following infection with lentiviruses, FOXP3-transduced cells were tested for potential immunosuppressive activities. ^3^H thymidine incorporation and CFSE dilution assays demonstrated that enforced expression of FOXP3 in CD3^+^CD56^+^ cells not only inhibited their own proliferative response to anti-CD3 stimulation but also that of CD4^+^CD25^–^ T cells (Fig. 4a,b). These results suggest that FOXP3 expression alone is sufficient to drive the differentiation of natural T cells into immunosuppressive cells.

TGF-β1 has been shown to induce FOXP3 expression in naïve T cells and their conversion into regulatory T cells^31^. Likewise, a large percentage of CD3^+^CD56^+^ cells acquired FOXP3 expression when stimulated with anti-CD3 plus anti-CD28 antibodies in the presence of TGF-β1 (Fig. 4c). Moreover, these cells exhibited a dose-dependent inhibitory effect on anti-CD3-induced proliferation of CD4^+^CD25^–^ T cells (Fig. 4d). Such an effect persisted with the addition of exogenous IL-2 but was abolished in transwell cultures (Fig. 4e), supporting that it is cell contact-dependent.

### Correlation of FOXP3-expressing CD3+CD56+ cells in the tumor and survival of HCC patients

**Figure 5:**
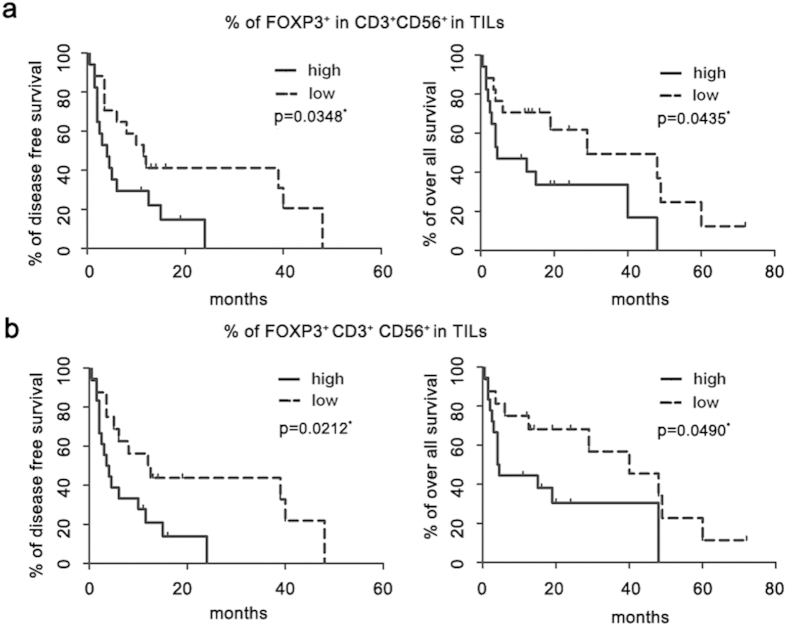
Increased intratumoral FOXP3-expressing CD3^+^CD56^+^ cells predicted poor survival of HCC patient. A total of 34 HCC patients were equally split into two groups according to either the percentage of FOXP3^+^ cells in CD3^+^CD56^+^ cells in TILs (**a**) or the percentage of FOXP3^+^CD3^+^CD56^+^ cells in TILs (**b**) respectively. Associations between FOXP3 expression and disease free survival or overall survival were estimated using Kaplan-Meier analysis.

The potent immunosuppressive activities of FOXP3-expressing CD3^+^CD56^+^ cells prompted an inquiry into the clinical relevance of their prevalence in tumor tissues. Due to lost to follow-up, only 34 of 41 HCC patients analyzed were available for prognostic analysis. A total of 34 HCC patients with complete clinical and experimental data were evenly divided into two groups on the basis of the median value of the percentage of FOXP3^+^ cells in the CD3^+^CD56^+^ population in TILs as the cut-off point (Supplementary Table S1). Kaplan-Meier analysis indicated that a high proportion of FOXP3^+^ cells was correlated with a poor outcome, both in terms of disease free survival and overall survival (Fig. 5a). As higher levels of FOXP3^+^ cells were frequently associated with greater reduction of CD3^+^CD56^+^ cells, to exclude the possibility that the survival disadvantage results from a reduced number of CD3^+^CD56^+^ cells. Kaplan-Meier analysis was performed by grouping patients according to the percentage of FOXP3^+^CD3^+^CD56^+^ cells in TILs. Again, increased intratumoral FOXP3^+^CD3^+^CD56^+^ cells predicted poor survival (Fig. 5b). The emergence of FOXP3^+^CD3^+^CD56^+^ cells in tumor tissues, therefore, may impose an adverse effect on anti-tumor immunity. Further, multivariate analysis revealed that the proportion of FOXP3^+^ cells in CD3^+^CD56^+^ cell population in TILs was an independent prognostic factor of overall survival (hazard ratio, 5.722; 95% confidence interval, 1.209–27.082; *P* < 0.05) (Table 2). The emergence of FOXP3^+^CD3^+^CD56^+^ cells in tumor tissues, therefore, may impose an adverse effect on anti-tumor immunity.

## Discussion

The present study identified a new subset of FOXP3^+^CD3^+^CD56^+^ cells occurring at a high frequency in the tumor tissue of HCC patients. Once acquiring FOXP3 expression, the cells lost the effector functions typically associated with hepatic CD3^+^CD56^+^ natural T cells. On the other hand, they displayed phenotypic and functional similarities to Treg cells. As a predominant lymphoid population in the liver, the CD3^+^CD56^+^ T cells are highly heterogeneous. In addition to Vα24 TCR-expressing iNKT cells, it contains a large proportion of T cells bearing αβ TCR of other specificities or even γδ TCR^26^,^27^. The comparable numbers of Vα24^+^ cells were observed among CD3^+^CD56^+^FOXP3^+^ versus CD3^+^CD56^+^FOXP3^–^ cells (Fig. 3a), suggesting that the hepatic natural T cells and iNKT cells are equally susceptible to the induction of FOXP3 expression. FOXP3-expressing human and mouse iNKT cells are also reported in several recent studies^32^,^33^,^34^. They can be induced *in vitro* by TGF-β and even *in vivo* following treatment with α-GalCer. Nevertheless, this is the first report of such cells spontaneously arising in human disease.

Over years, multiple immune suppressor cells are found to be accumulated in HCC patients^12^. The relative contribution of each cell type to tumor development and progression, however, remains to be evaluated. Given the overall abundance of CD3^+^CD56^+^ T cells in the liver and the frequent occurrence of FOXP3 expression (up to 65%) in the tumor tissue, the FOXP3^+^CD3^+^CD56^+^ cells could play a substantial role in the immunosuppressive network in HCC, at least for some patients. In line with this speculation, the prevalence of FOXP3^+^CD3^+^CD56^+^ cells in TILs was found to be inversely correlated with patient survival. Several reports have also indicated that an increased intratumoral FOXP3-positive cells was associated with decreased overall survival of HCC patients^35^,^36^,^37^,^38^. But all these studies applied immunohistochemistry staining to assess FOXP3^+^ lymphocytes of HCC tissues, and further analysis for subpopulations of intratumoral FOXP3^+^ lymphocytes was not performed. In our study, we quantified the intratumoral FOXP3-expressing cells in CD3^+^CD56^+^ cell population in HCC tissues by flow cytometry, and clearly demonstrated that intratumoral FOXP3^+^CD3^+^CD56^+^ cells was associated with DFS and OS in HCC patients. As hepatic environment was characterized by the presence of a large number of CD3^+^CD56^+^ cells, our findings provided a possibility that FOXP3^+^CD3^+^CD56^+^ cells among the intratumoral FOXP3^+^ cells may be the most important cell population playing suppressive roles in the progression of HCC. Of course, it needs further detailed investigation on different intratumoral FOXP3^+^ populations in HCC tissues.

Despite the high incidence of FOXP3^+^CD3^+^CD56^+^ cells in HCC, we found no evidence for FOXP3 expression by CD3^+^CD56^+^ cells in normal liver or peripheral blood of healthy donors. Even for HCC patients, the FOXP3-expressing CD3^+^CD56^+^ cells were only detected in tumor tissues but not in circulation. Therefore, these cells are most likely derived from hepatic natural T cells infiltrating the tumor tissues. TGF-β has been shown to be a potent inducer of FOXP3 expression in CD4^+^ naive T cells and iNKT cells^31^,^32^,^33^. A similar effect was observed with CD3^+^CD56^+^ cells in culture. Exposure to TGF-β1 not only induced high levels of FOXP3 expression, but also conferred an immunosuppressive activity. Conflicting data, however, are reported about the expression of TGF-β1 in HCC. While elevated levels of TGF-β1 are documented in some reports^39^,^40^, we found reduced expression of TGF-β1 mRNA in tumor versus adjacent non-tumor tissues^28^. Although the reason for the discrepancy remains elusive, it questions to what extent TGF-β1 is responsible for the accumulation of FOXP3^+^CD3^+^CD56^+^ cells in the tumor tissue. In consideration of the co-existence of multiple subsets of immune suppressor cells in HCC, it would also be interesting to examine whether one would induce another. The recent report by Hoechst *et al*. provides a good example, in which they demonstrate that CD14^+^HLA-DR^low/neg^ immature myeloid cells are capable of converting CD4^+^ T cells into Tregs in co-cultures^18^.

Multiple strategies are engaged by regulatory immune cells to exert their immunosuppressive functions, including suppression by inhibitory cytokines like IL-10 and TGF-β, suppression by perforin and granzyme-induced cytolysis, suppression by metabolic disruption, and suppression by targeting DCs through CTLA-4 and IDO^41^,^42^. Although the exact mechanisms of immunosuppression by FOXP3^+^CD3^+^CD56^+^ cells is yet to be defined, it is clearly cell contact-dependent, as physical separation from responder cells using transwell abolished the anti-proliferative activity of these cells. The cell contact-dependence argues against any substantial role of soluble inhibitory factors. The implication of cytolysis is also largely excluded in view of the mutually exclusive expression of FOXP3 versus perforin, one molecule thought to be critical for cytolysis by Treg cells^43^. Besides, we found that addition of exogenous IL-2 to the assay had no impact on the inhibitory effect, suggesting that IL-2 consumption is an unlikely mechanism either. Given the clear evidence of cell contact-dependence, future studies will be concentrated on the role of surface molecules, including the various NK receptors.

In conclusion, the present study reported for the first time a subset of FOXP3^+^CD3^+^CD56^+^ cells spontaneously arising in the tumor mass of HCC patients. These cells, which are most likely derived from CD3^+^CD56^+^ cells heavily populating the liver, imposed a potent and cell contact-dependent inhibition on T cell activation. Moreover, their prevalence in the tumor predicates worse outcome for HCC patients. The emergence of this new subset of regulatory cells further highlights the complexity of the immunosuppressive network in HCC. In addition, they may represent a previously unrecognized target of immunotherapy for HCC.

## Methods

### Patients and healthy donors

A total of 48 HCC patients seen at the Center of Hepatobiliary Surgery, Peking University People’s Hospital (Beijing, China) were enrolled in the present study. HCC was diagnosed according to the diagnostic guidelines of the European Association for the Study of the Liver. Patient characteristics and demographic data are shown in Table 1. Tumor and non-tumor (>5 cm from the tumor margin) tissues were collected at the time of surgery. Also collected were blood samples from 11 of these patients. As controls, liver specimens were obtained from 4 patients with hepatic benign hemangioma at the same center, and blood samples from 11 healthy donors at the blood bank of Beijing Red Cross. Collection of the samples was approved by Hospital Ethic Review Committee of Peking University People’s Hospital and agreed by the patients with written consent in accordance with relevant approved guidelines.

### Cell isolation and cell sorting

Single cell suspensions of mononuclear cells (MNC) from freshly dissected tumor and non-tumor tissues were prepared by mechanical dissociation and collagenase treatment as described previously^28^. Tumor-infiltrating lymphocytes (TILs), non-tumor-infiltrating lymphocytes (NILs), and peripheral blood mononuclear cells (PBMC) were isolated by centrifugation on Ficoll density gradients. Cells were either immediately used for experiments or cryopreserved for future use. To purify the CD3^+^CD56^+^, CD3^+^CD56^+^CD25^+^, CD4^+^CD25^high^ and CD4^+^CD25^–^ subsets, TILs, NILs or PBMC were stained with the appropriate combinations of fluorescence labeled antibodies and then fractionated using BD FACSAria (BD Biosciences, San Diego, CA) with a purity of 95–98%.

### Antibodies and flow cytometry

Flow cytometry was performed to analyze the phenotype and frequency of various subpopulations in TILs, NILs and PBMC using a series of fluorescence labeled monoclonal antibodies specific for CD3, CD4, CD8, CD25, CD56, CTLA-4, CD45RO, CD127, αβTCR, TCR Vα24, γδTCR, IFN-γ, perforin with appropriate isotype-matched controls. Anti-TCR Vα24-FITC was obtained from Beckman Coulter (Beckman Coulter Inc, Brea, CA). Antibodies against CD45RO, CTLA-4, and CD127 were purchased from eBioscience (San Diego, CA), and the rest from BD Pharmingen (San Diego, CA). PBS57 loaded and unloaded control mCD1d tetramers were kindly provided by the tetramer facility of NIH. Staining for FOXP3 protein was performed using the FOXP3 kit (eBioscience) according to the manufacturer’s instructions. For intracellular staining of IFN-γ and perforin, cells were first stimulated with 50 ng/ml phorbol-12-myristate-13-acetate (PMA, Sigma-Aldrich, Saint Louis, MO) and 1 μg/ml ionomycine (Sigma-Aldrich) for 4 hours with the addition of 10 μg/ml brefeldin A (BFA, Sigma-Aldrich) in the last 2 hours. After surface staining, cells were fixed and permeabilized using CytoFix/Cytoperm (BD Pharmingen), followed by staining for intracellular antigens. Data were acquired using FACSCalibur (BD Biosciences) and analyzed using the CellQuest software (BD Biosciences, San Diego, CA, USA).

### Expansion of CD3^+^CD56^+^ cells

To obtain a sufficient number of cells for lentiviral transduction, CD3^+^CD56^+^ cells purified from NILs were cultured and expanded in RPMI 1640 medium supplemented with 10% human AB serum (GIBCO, Invitrogen, Carlsbad, CA), and stimulated with soluble anti-CD3 (1 μg/ml, BD Pharmingen), soluble anti-CD28 (1 μg/ml, BD Pharmingen) and rhIL-2 with rhIL-15 (10 ng/ml, R&D system, Inc, Minneapolis, MN) in the presence of allogeneic irradiated PBMC as antigen presenting cell (APC) for three weeks to get expansion with the maintenance of CD3 and CD56 coexpression phenotype. The medium was replaced with fresh RPMI 1640 with the addition of 10% AB human serum and rhIL-2 and rhIL-15 at a final concentration of 10 ng/ml every three days. Sixteen hours before lentiviral transduction, the culture was shifted to X-VIVO15 medium (BioWhittaker, Lonza Walkersville, MD, USA) containing 10 ng/ml rhIL-2, 10 ng/ml rhIL-7 (R&D system), and 1 μg/ml soluble anti-CD3 with the addition of allogeneic irradiated APCs at a ratio of 1:5.

### Lentivirus package and lentiviral transduction of CD3^+^CD56^+^ cells

Enforced expression of FOXP3 in CD3^+^CD56^+^ cells was achieved through lentiviral transduction. Viral particles were produced by co-transfection of HEK 293T cells with pSPAX2, pVSVG plus the bidirectional lentiviral vector pCCL.FP3 expressing human FOXP3 and △NGFR^44^ or the empty vector pCCL, all of which were kindly provided by Dr. Megan K Levings (University of British Columbia, Canada). The culture supernatant was collected 48 hours afterwards and the viral titer was determined through transduction of 293T cells with successive dilutions of the supernatant.

*In vitro* expanded CD3^+^CD56^+^ cells were infected with FOXP3-expressing lentiviruses at a MOI (multiplicity of infection) of 10 on Retronectin (Takara Bio Inc, OtsuShiga, Japan) coated plates in the presence of 5 μg/ml PolyBrene (sigma-Aldrich, Oakville, Canada). Following lentiviral transduction, cells were continually cultured in complete medium supplemented with rhIL-2 (10 ng/ml) and rhIL-15 (10 ng/ml) for 10–14 days with medium replacement every three days. Before being used in functional assay, the cells were allowed to rest overnight in rhIL-2 and rhIL-15 free medium.

### T cell proliferation and suppression assay

Purified CD4^+^CD25^–^ cells (responder cells) were either directly plated or preloaded with carboxyfluorescein diacetate succinimidyl ester (CFSE) and then seeded into 96-well round-bottomed plates (Corning Costar Corp, NY) in triplicate at a density of 5 × 10^4^ cells/well. Cell proliferation was induced by stimulation with anti-CD3 (1 μg/ml) in the presence of irradiated allogeneic PBMCs as APC (2×10^5^/well, irradiated at 35 Gy) for 4 days. Proliferation was measured either by monitoring CFSE dilution or by thymidine incorporation, in which ^3^H thymidine (0.5 μCi/well, Amersham, Freiburg, Germany) was added to the culture 10 hours prior to cell harvesting.

To observe the inhibitory effect on cell proliferation, CD25^+^CD3^+^CD56^+^ cells directly purified from TILs, FOXP3-transduced CD3^+^CD56^+^ cells, TGF-β1-treated CD3^+^CD56^+^ cells, or CD4^+^CD25^high^ conventional Treg cells purified from PBMC were added to the assays at different ratios to responder cells from the same donor. Relative suppression was calculated as: % suppression = [1 – % of proliferation in coculture/% of proliferation in responder cells culture alone] × 100%. In some settings, the cells to be tested were separated from responder cells using transwell (0.4 μm, Corning Costar Corp). In other settings, exogenous rhIL-2 was added to the culture at a concentration of 10 ng/ml.

### Induction of FOXP3 expression in CD3^+^CD56^+^ cells by TGF-β1

Sorted CD3^+^CD56^+^ cells from NILs were activated with soluble anti-CD3 plus anti-CD28 (1 μg/ml for each) in the presence or absence of TGF-β1 (10ng/ml, R&D system). At Day 4, cells were harvested, and examined for FOXP3 expression using flow cytometry and for anti-proliferative activities through CFSE dilution assay.

### Statistical analysis

Student’s t test was used to determine the significance of the difference between group means using GraphPad Prism software (Graphpad, La Jolla, CA, USA). The follow-up time of a total of 34 HCC patients were calculated as the interval between the date of surgery treatment and the last follow-up or death. The log-rank test was applied to assess the difference between survival curves by Kaplan-Meier method using GraphPad Prism software. The Cox proportional hazards model was used for multivariate analyses using SPSS version 12.0. *P* < 0.05 was considered with statistically significant difference.

### Ethical approval

This study was approved by the Hospital Ethic Review Committee of Peking University People’s Hospital and was performed in accordance with the approved ethical standards laid down in the 1964 declaration of Helsinki and all subsequent revisions.

## Additional Information

**How to cite this article**: Li, X. *et al*. Identification of a FOXP3^+^CD3^+^CD56^+^ population with immunosuppressive function in cancer tissues of human hepatocellular carcinoma. *Sci. Rep*. **5**, 14757; doi: 10.1038/srep14757 (2015).

## Supplementary Material

Supplementary Information

## Figures and Tables

**Table 1 t1:** Clinical characteristics of 48 patients.

Variable	Results
Sex (male/female)	35/13
Age[year,median(range)]	60(36–84)
α-fetoprotein level [ng/ml,median (range)]	336 (0–1,210)
HBs Ag positivity (%)	65.20%
HCV antibody positivity (%)	6.50%
TNM stage (I/II/III/IV)	8/17/16/7
Differentiation(high/medium/low)	27 7 14

Abbreviation: HBs Ag: hepatitis B surface antigen; HCV: hepatitis C virus; TNM: tumor-node-metastasis.

**Table 2:** Multivariate analysis of parameters associated with disease free survival and overall survival.

Variable	Disease free survival			Overall survial		
	HR	95% CI	P	HR	95% CI	P
Age (year, >60 *vs* ≦60)	1.232	0.502–3.029	0.649	1.305	0.478–3.563	0.604
Sex (male *vs* female)	1.046	0.417–2.620	0.924	1.554	0.526–4.589	0.425
TNM stage (III/IV *vs* I/II)	1.440	0.516–4.014	0.486	2.246	0.717–7.041	0.165
AFP (ng/ml, >300 *vs* ≦ 300)	1.095	0.386–3.106	0.865	0.544	0.186–1.589	0.265
Intratumoral TILs (high *vs* low)						
% of FOXP3^+^ in CD3^+^CD56^+^	1.940	0.602–6.247	0.267	5.722	1.209–27.082	**0.028**
% of FOXP3+CD3+CD56+	1.900	0.645–5.597	0.244	0.605	0.152–2.397	0.474

Abbreviation: HR: hazard ratio; 95% CI: 95% confidence interval; P: p value.
