# Supplementary material for: Identification of a FOXP3+CD3+CD56+ population with immunosuppressive function in cancer tissues of human hepatocellular carcinoma
Source: Sci Rep. 2015 Oct 6;5:14757. doi: 10.1038/srep14757 (PMC4594002; doi:10.1038/srep14757)
Supplement: Supplementary Information [file srep14757-s1.pdf]

Identification of a $FOXP3^{+}CD3^{+}CD56^{+}$ population with immunosuppressive function in cancer tissues of human hepatocellular carcinoma

Xiaofeng Li, Jirun Peng, Yanli Pang, Sen Yu, Xin Yu, Pengcheng Chen, Wenzhen Wang, Wenling Han, Jun Zhang, Yanhui Yin, Yu Zhang

Table S1 Characteristics of 34 patients in survival analysis

Patient No.	Gender	Age (year)	AFP (ng/ml)	TNM (stage)	Survival time (month)	Disease free time (month)	Relative levels of specific subsets: % of $FOXP3^{+}$ in $CD3^{+}CD56^{+}$	Relative levels of specific subsets: % of $CD3^{+}CD56^{+}FOXP3^{+}$
1	male	73	1210	II	4.5	4.5	high	high
2	male	64	4.21	I	12.5	12.5	high	low
3	male	47	3.29	III	>24	11.5	low	high
4	male	77	4.36	II	>24	12	low	low
5	male	70	9	II	48	39	low	low
6	male	57	12	II	60	48	low	low
7	male	65	1210	II	49	40	low	low
8	female	54	8.58	II	>72	48	low	low
9	male	70	16.52	III	19	10	low	high
10	female	58	3.03	II	1.5	1.5	low	low
11	female	64	1210	II	4	2	high	high
12	male	54	1210	II	15	6	high	high
13	female	57	362.9	IV	40	5	high	low
14	male	66	69.35	I	29	6	low	low
15	female	65	1210	III	1.5	1.5	high	high
16	male	80	270.5	II	>24	24	high	high
17	male	47	1210	IV	3	3	high	high
18	male	39	1210	II	4	3.5	low	high
19	male	36	1210	II	2.5	2.5	high	high
20	female	77	1.89	III	0.5	0.5	low	low
21	male	57	139.5	IV	0.5	0.5	high	high
22	male	69	126	I	6	3.5	low	low
23	male	37	7.55	II	3.5	3.5	low	low
24	male	84	1210	II	4	4	high	high
25	male	63	1210	IV	1.5	1.5	high	high
26	male	52	1210	I	>20	2	high	high
27	female	46	40.54	IV	>16	>16	low	high
28	male	65	36.5	I	48	15	high	high
29	female	75	3.99	I	>19	>19	high	low
30	male	41	1210	IV	>13	>13	low	low
31	female	57	1.51	II	>12	8	low	low
32	male	49	52.28	II	>11	>11	high	high
33	female	65	2.04	II	>14	>14	low	low
34	male	55	15.84	IV	2	2	high	high
